# The relationship between depression and benign prostatic hyperplasia in middle-aged and elderly men in India: a large-scale population study

**DOI:** 10.1186/s12889-023-17027-2

**Published:** 2023-11-03

**Authors:** Xiaoyang Liu, Kai Ma, Luchen Yang, Zhufeng Peng, Pan Song, Zhenghuan Liu, Jing Zhou, Yunfei Yu, Qiang Dong

**Affiliations:** Department of Urology, Institute of Urology, West China Hospital, Sichuan University, Chengdu, China

**Keywords:** Benign prostatic hyperplasia, Depression, Elderly, LASI, Risk factors

## Abstract

**Background:**

There have been few investigations on the association between depression and benign prostatic hyperplasia (BPH). This study aims to explore the correlation between depression and BPH among middle-aged and older men in India.

**Methods:**

We utilized data from male individuals aged 45 years and older who participated in the initial wave (2017–2018) of the Longitudinal Aging Study in India (LASI). The presence of BPH symptoms was based on self-reported information, while depressive symptoms were evaluated using CESD-10. The analysis was a cross-sectional study conducted on a final sample size of 30,108 male participants. To examine associations, we employed multivariate logistic regression analysis along with subgroup analysis and interaction tests.

**Results:**

A total of 439 (1.46%) men reported BPH and had a higher depression score (10.18 ± 4.22 vs. 9.28 ± 4.00). The findings indicated a significant association between the depression score and the likelihood of developing BPH, even after accounting for all potential confounding variables (OR = 1.054, 95% CI: 1.030–1.078, *p* < 0.00001). The participants were then categorized into a depression group and a normal group based on their CESD-10 score, using a threshold of 10 to ascertain the existence or nonexistence of depression. After adjusting for all variables in model IV, the findings continued to exhibit statistical significance (OR = 1.611, CI: 1.327–1.955, *p* < 0.00001). Significant interaction effects of age, education level, caste or tribe, and alcohol consumption were observed (*p* for interaction < 0.05).

**Conclusion:**

Our research found that BPH was significantly linked to the presence of depressive symptoms among middle-aged and elderly Indian men. Additional prospective research is necessary to clarify this association and investigate potential mechanisms.

**Supplementary Information:**

The online version contains supplementary material available at 10.1186/s12889-023-17027-2.

## Introduction

Benign prostatic hyperplasia (BPH), characterized by urinary retention and incontinence, is the most prevalent urologic condition in elderly men. The occurrence of BPH often manifests once male individuals reach the age of 40 and exhibits an upward trend as time progresses. Specifically, it impacts around 25% of males in their 50 s, 33% of males in their 60 s, and 50% of males in their 80 s [1, 2]. Bladder Outlet Obstruction occurs when the prostate physically compresses the urethra as a consequence of an increase in stromal cells and prostate epithelial cells, which also led to lower urinary tract symptoms (LUTS) including obstructive voiding symptoms and irritable voiding symptoms [3].

Depression is a significant global health concern, posing a substantial danger to the overall welfare of about 350 million individuals globally, with a special emphasis on elderly people [4]. Based on pertinent academic investigations, it has been observed that depression has a negative impact on cognitive functioning and overall quality of life. Additionally, individuals affected by depression have an elevated possibility of developing chronic comorbidities, including cardiovascular disease and diabetes [5, 6]. Therefore, it is crucial to investigate the prospective geriatric diseases associated with depression.

Various risk factors, including metabolic syndrome, overweight, sex hormone levels, and inflammation, have been recognized as contributors to the higher prevalence of BPH [3]. In addition, depression may have an impact on the body's hormone metabolism and systemic inflammation [7–9]. Previous research has shown a notable association between the level of severe depressive symptoms and a decline in overall testosterone levels within the male bloodstream [10]. And depression may activate fundamental inflammatory signaling networks that might lead to the proliferation of prostatic cells, subsequently causing hyperplasia of the prostate. The potential influence of depression on prostate health may be attributed to changes in circadian rhythm control, fluctuations in sex hormone levels, and modifications in nervous system feedback mechanisms. Both of these illnesses are frequently observed in elderly males, but their association remains obscure. The negative effects of BPH may contribute to depression, and vice versa, depression may trigger the development of BPH in the mechanism mentioned previously [11]. Limited research has provided a few possibilities. Xiong found that depression may be associated with LUTS suggestive of BPH in a study involving 5125 participants [11]. Despite this, insufficient evidence exists to elucidate the association between BPH and depression. Therefore, the objective of this investigation was to investigate any potential associations between depression and the occurrence of BPH. As one of the illnesses impacting the quality of life of middle-aged and older men, the widespread incidence of BPH exacerbates their suffering as well as the economic and medical burden on our society [12]. Our results might help to create new approaches to avoiding BPH and improving the quality of life for elderly people.

## Method

### Data resource

**Fig. 1:**
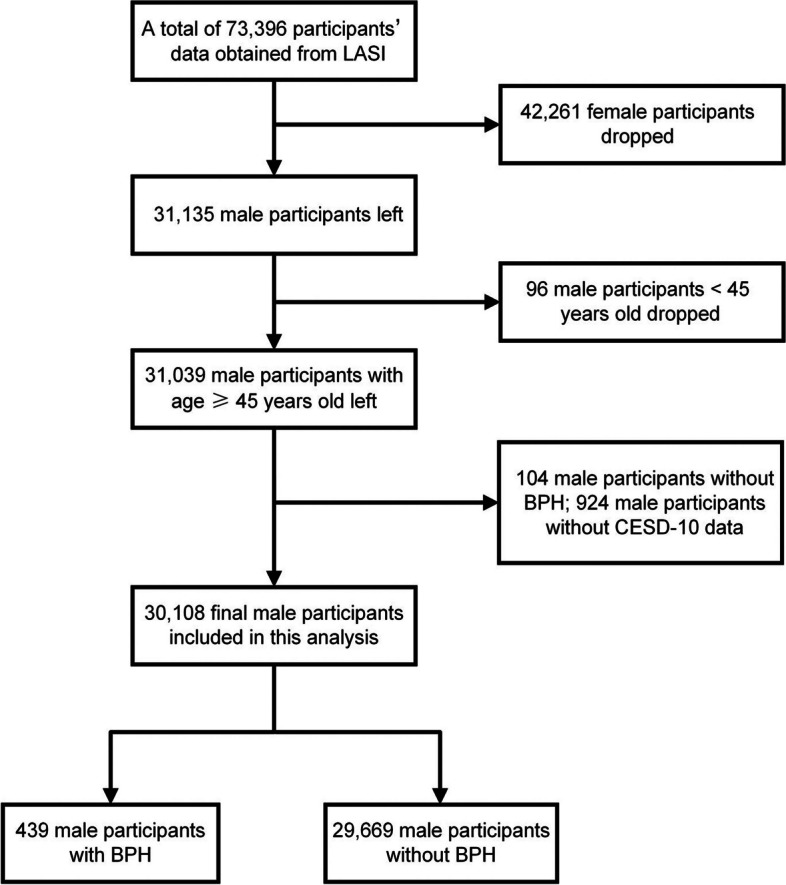
Flowchart of the participants selection

The data used in this study were sourced from LASI Wave 1, a nation-wide comprehensive study of the psychological, physiological, social, and economic wellbeing of elderly adults in India, harmonized with the worldwide Health and Retirement Study and its sister studies (https://g2aging.org/). The LASI Wave 1 included a total of 73,396 individuals aged 45 years and older, representing India and all its 35 states or union territories except the state of Sikkim. LASI adopted a multistage stratified area probability cluster sampling design. Within each state, a three-stage sampling design in rural areas and a four-stage sampling design in urban areas were followed to select households for the study. Specifically, the first stage involved the selection of a primary sampling unit, and the second stage involved the selection of villages in rural areas and wards in urban areas in the selected primary sampling units. In rural areas, households were selected from selected villages in the third stage. However, sampling in urban areas involved an additional stage in the third stage, namely one census enumeration block, which was randomly selected in each urban ward. And in the fourth stage, households were selected from this census enumeration block. In our study, female individuals were excluded because prostatic hyperplasia is a male disease, and participants without data on the benign prostatic hyperplasia and depression scales were also excluded. Figure 1 illustrates the overall number of individuals that were enrolled in the research. All participants provided their formal assent by signing a recorded consent form, the specifics of which may be seen on the official LASI Wave 1 website.

### Definition of depression and BPH

Symptoms of depression were obtained through a questionnaire as the outcome. The depressive signs and symptoms of the participants were assessed using the CESD-10, a shortened version of the CESD-20, which is a widely used tool for evaluating depression symptoms in the general population [13]. Ten CESD-10 questions were used to assess the depressive symptoms of participants in the LASI study. Respondents were rated “During the past week, was the following true for you much of the time” on 4 levels, namely “Rarely or never (less than 1 day)”;” Sometimes (1 or 2 days)”; “Often (3 or 4 days)”; “Most or all of the time (5–7 days)”, which corresponds to a score of 0–3 according to the question. The participants were categorized into groups based on their CESD-10 score, using a threshold of 10 to ascertain the existence or nonexistence of depression (individuals with a depression score below 10 were classified as having symptoms of depression) [14].

Benign prostate hyperplasia was self-reported and evaluated by asking the following question: "Have you ever been diagnosed with any of the following urogenital conditions or diseases with options including BPH?".

### Covariates

In order to account for any potential confounding variables, this study included specific parameters that have been shown to influence the development of BPH. The age was quantified in units of years. Education was classified into four categories: above higher secondary, secondary and higher secondary, middle school, or under and never educated. In light of India's unique ethnic structure, the population has been categorized into no or other castes, other backward class, scheduled tribe, and those belonging to scheduled caste. In terms of marital status, there were three categories: widowed, married or partnered, and others. Residential locations were classified into two distinct categories: rural and urban. Underweight (< 18.5 kg/m2), normal (18.5–25 kg/m2), and overweight (> 25 kg/m2) BMI categories were established. On the basis of frequency, classifications were made for consumption of alcohol (heavy, moderate, light, never), physical activity frequent, sometimes, seldom, never), and consumption of cigarettes (heavy, moderate, light, never). Furthermore, we identified many chronic disorders as potential confounding factors, based on the inquiry: "Has a health professional ever diagnosed you with any of the following chronic conditions or diseases?". This involved prior medical diagnoses such as hypertension, hyperlipidemia, malignancy, cardiovascular diseases, respiratory illness, cerebrovascular accidents, musculoskeletal disorders, and any neurological disorders. The missing covariate variables were imputed using multivariate imputation techniques that rely on predictive mean matching approaches.

### Statistical analysis

Standard deviations and means are used to represent continuous variables in the baseline characteristics, whereas rates and percentages are utilized to illustrate categorical variables. The Kruskal–Wallis test was utilized for determining *p*-values for continuous variables, without requiring the data to conform to assumptions of normality and homogeneity of variance. In contrast, chi-square tests were employed to analyze categorical data. The Fisher exact test was applied when the theoretical number < 10 [15].

The depression score was initially treated as a continuous variable. Then, since we used a CESD-10 total score threshold of 10 to determine the existence or nonexistence of depressive symptoms, we divided the participants into two groups: the normal group and the depression group. We utilized multivariate logistic regression analysis to investigate the relationship between depression and BPH, adjusting for a number of covariates to reduce the influence of additional confounding variables on the investigation's outcomes. Model I was adjusted merely for age groups. Model II, on the other hand, was adjusted for age group, caste/tribe, residence, marital status, and education level. Additionally, Model III added variables including tobacco consumption, alcohol consumption, and physical activity to Model II. In the present study, Model IV was adjusted for additional various covariates including BMI, hypertension, diabetes, high cholesterol, tumor, chronic lung disease, chronic heart disease, stroke, bone or joint disease, and neurological or psychiatric disorder. We conducted interaction analyses to examine the heterogeneity of the association between depression and BPH stratified by covariates. The participants were categorized into four unique groups based on age cutoffs of 60, 70, and 80. Subgroup analyses were conducted using stratified logistic regression models, and the *p*-value for interaction was determined using the log-likelihood ratio test, which involved comparing models with and without covariate interactions. A significance level of 0.05 was considered to be statistically significant for all the statistical findings in this study. All analyses were performed using R version 4.2.2.

## Results

### Baseline characteristics

There are a total of 30,108 participants in the study, including 17,991 participants with no depressive symptoms and 12,117 participants with depressive symptoms and individuals’ baseline characteristics was listed on Table 1. At baseline level, there is evidence to suggest that males diagnosed with benign prostatic hyperplasia (BPH) exhibit elevated levels of depression compared to those who do not have (depression score: 10.18 ± 4.22 vs. 9.28 ± 4.00), and participants with BPH are more likely to have depressive symptoms (52.16% vs. 40.07%). Simultaneously, it was observed that the BPH group had a higher average age compared to the non-BPH group (63.97 ± 10.99 vs. 59.94 ± 10.57). Furthermore, there were statistically significant differences (*P* < 0.001) in caste/tribe, educational levels, alcohol consumption, hypertension, diabetes, high cholesterol, chronic heart disease, and bone or joint disease between the group with BPH and without.

**Table 1:** Baseline characteristics of the participants selection (BPH: benign prostatic hyperplasia, SD: standard deviation, BMI: Body Mass Index Mean, SD for continuous variables: *P* value was calculated by Kruskal–Wallis rank-sum test, Number (%) for categorical variables: *P* value was calculated by chi-square test

		**BPH**		
		**No**	**Yes**	***P***
		***n* = 29,669**	***n* = 439**	
Age (mean ± (SD))		59.94 ± 10.57	63.97 ± 10.99	< 0.001
Depression score (mean ± (SD))		9.28 ± 4.00	10.18 ± 4.22	< 0.001
Age class (%)	< 60	15,168 (51.12)	155 (35.31)	< 0.001
	< 70	8691 (29.29)	147 (33.49)	
	< 80	4357 (14.69)	91 (20.73)	
	> = 80	1453 (4.90)	46 (10.48)	
Depression class (%)	< 10	17,781 (59.93)	210 (47.84)	< 0.001
	> = 10	11,888 (40.07)	229 (52.16)	
Caste/tribe (%)	Scheduled caste	4857 (16.49)	68 (15.56)	< 0.001
	Scheduled trible	5271 (17.90)	36 (8.24)	
	Other backward class	11,332 (38.48)	158 (36.16)	
	No or other caste	7990 (27.13)	175 (40.05)	
Residence (%)	Rural	19,477 (65.65)	277 (63.10)	0.287
	Urban	10,192 (34.35)	162 (36.90)	
Marital status (%)	Married	26,049 (87.80)	379 (86.33)	0.212
	Widowed	2662 (8.97)	49 (11.16)	
	Others	957 (3.23)	11 (2.51)	
Education level (%)	Never	9231 (31.11)	115 (26.20)	< 0.001
	Middle school or under	12,375 (41.71)	156 (35.54)	
	Secondary and higher secondary	5774 (19.46)	122 (27.79)	
	Above higher secondary	2289 (7.72)	46 (10.48)	
BMI (%)	underweight	5182 (19.09)	61 (14.70)	0.077
	normal	15,438 (56.87)	249 (60.00)	
	overweight	6525 (24.04)	105 (25.30)	
Tobacco consumption (%)	Never	13,230 (44.63)	188 (42.92)	0.205
	light	8145 (27.48)	109 (24.89)	
	moderate	6332 (21.36)	105 (23.97)	
	vigorous	1937 (6.53)	36 (8.22)	
Alcohol consumption (%)	Never	19,607 (66.13)	276 (63.16)	< 0.001
	light	5721 (19.30)	118 (27.00)	
	moderate	2885 (9.73)	29 (6.64)	
	heavy	1435 (4.84)	14 (3.20)	
Physical activity (%)	never	14,945 (50.41)	235 (53.65)	0.057
	seldom	2851 (9.62)	50 (11.42)	
	sometimes	2521 (8.50)	41 (9.36)	
	frequent	9332 (31.47)	112 (25.57)	
Hypertension (%)	no	22,331 (75.28)	278 (63.33)	< 0.001
	yes	7331 (24.72)	161 (36.67)	
Diabetes (%)	no	25,757 (86.85)	349 (79.50)	< 0.001
	yes	3901 (13.15)	90 (20.50)	
High cholesterol (%)	no	28,748 (96.91)	409 (93.17)	< 0.001
	yes	918 (3.09)	30 (6.83)	
Tumor (%)	no	29,515 (99.49)	433 (98.63)	0.031
	yes	150 (0.51)	6 (1.37)	
Chronic lung disease (%)	no	27,885 (94.00)	400 (91.12)	0.016
	yes	1781 (6.00)	39 (8.88)	
Chronic heart diseases (%)	no	28,445 (95.88)	404 (92.03)	< 0.001
	yes	1221 (4.12)	35 (7.97)	
Stroke (%)	no	29,030 (97.86)	430 (97.95)	1
	yes	636 (2.14)	9 (2.05)	
Bone or joint diseases (%)	no	26,370 (88.89)	349 (79.50)	< 0.001
	yes	3296 (11.11)	90 (20.50)	
Neurological or psychiatric problem (%)	no	29,026 (97.86)	423 (96.36)	0.048
	yes	636 (2.14)	16 (3.64)	

### Association between depression and benign prostatic hyperplasia

The results of multivariate logistic regression analyses examining the association between depression and the likelihood of developing BPH among study participants are shown in Table 2. In the crude model, the correlation between depression symptoms and the likelihood of BPH indicated that elderly individuals with a high depression score had an increased risk of developing BPH (OR: 1.631; 95% CI: 1.351–1.970, *P* < 0.00001). After controlling for age, the odds ratio (OR) of Model I was found to be 1.557 (CI: 1.288–1.882, *p* < 0.00001). The link between the variables in Model II (OR: 1.637; 95% CI: 1.352 -1.982, *p* < 0.00001) and Model III (OR: 1.656; 95% CI: 1.367 -2.007, *p* < 0.00001) remained consistent even after accounting for several possible confounding factors. After adjusting for all variables in model IV, the findings continued to exhibit statistical significance (OR = 1.611, CI: 1.327—1.955, *p* < 0.00001). Based on the findings of Model I in the regression analysis, it can be inferred that age potentially serves as a risk factor influencing the association between depression and BPH. It follows from Model III that physical activity, alcohol consumption, and cigarette consumption may all contribute to this connection. The findings of Model IV indicate that the presence of comorbidities significantly influences the overall relevance. Besides, when the CESD-10 score was analyzed as a continuous variable, the results of Model IV revealed a statistically significant association between depression and BPH after adjusting for all potential confounding variables (OR = 1.054, 95% CI: 1.030–1.078, *p* < 0.00001).

**Table 2:** Multivariate regression model of the relationship between depression and the risk of benign prostatic hyperplasia–sensitivity analysis

Exposure	Crude model		Model I		Model II		Model III		Model IV	
	OR (95%CI)	*P*-value	OR (95%CI)	*P*-value	OR (95%CI)	*P*-value	OR (95%CI)	*P*-value	OR (95%CI)	*P*-value
Depression score	1.054 ( 1.031, 1.077)	*p* < 0.00001	1.048 ( 1.025, 1.071)	0.00004	1.055 ( 1.032, 1.079)	*p* < 0.00001	1.058 ( 1.035, 1.082)	*p* < 0.00001	1.054 ( 1.03, 1.078)	*p* < 0.00001
Depression class	1.631 ( 1.351, 1.97)	*p* < 0.00001	1.557 ( 1.288, 1.882)	*p* < 0.00001	1.637 ( 1.352, 1.982)	*p* < 0.00001	1.656 ( 1.367, 2.007)	*p* < 0.00001	1.611 ( 1.327, 1.955)	*p* < 0.00001

Crude model adjust for none

Model I adjust for: age

Model II adjust for: age; caste; residence; marital status; education level

Model III adjust for: age; caste; residence; marital status; education level; tobacco consumption; alcohol consumption; physical activity

Model IV adjust for: age; caste; residence; marital status; education level; tobacco consumption; alcohol consumption; physical activity; BMI; Hypertension; Diabetes; High cholesterol; Tumor; Chronic lung disease; Chronic heart diseases; Stroke; Bone or joint diseases; Neurological or psychiatric problem

*OR* Odd ratio, *CI* Confidence interval, *BMI* Body mass index

### Subgroup analysis

**Fig. 2:**
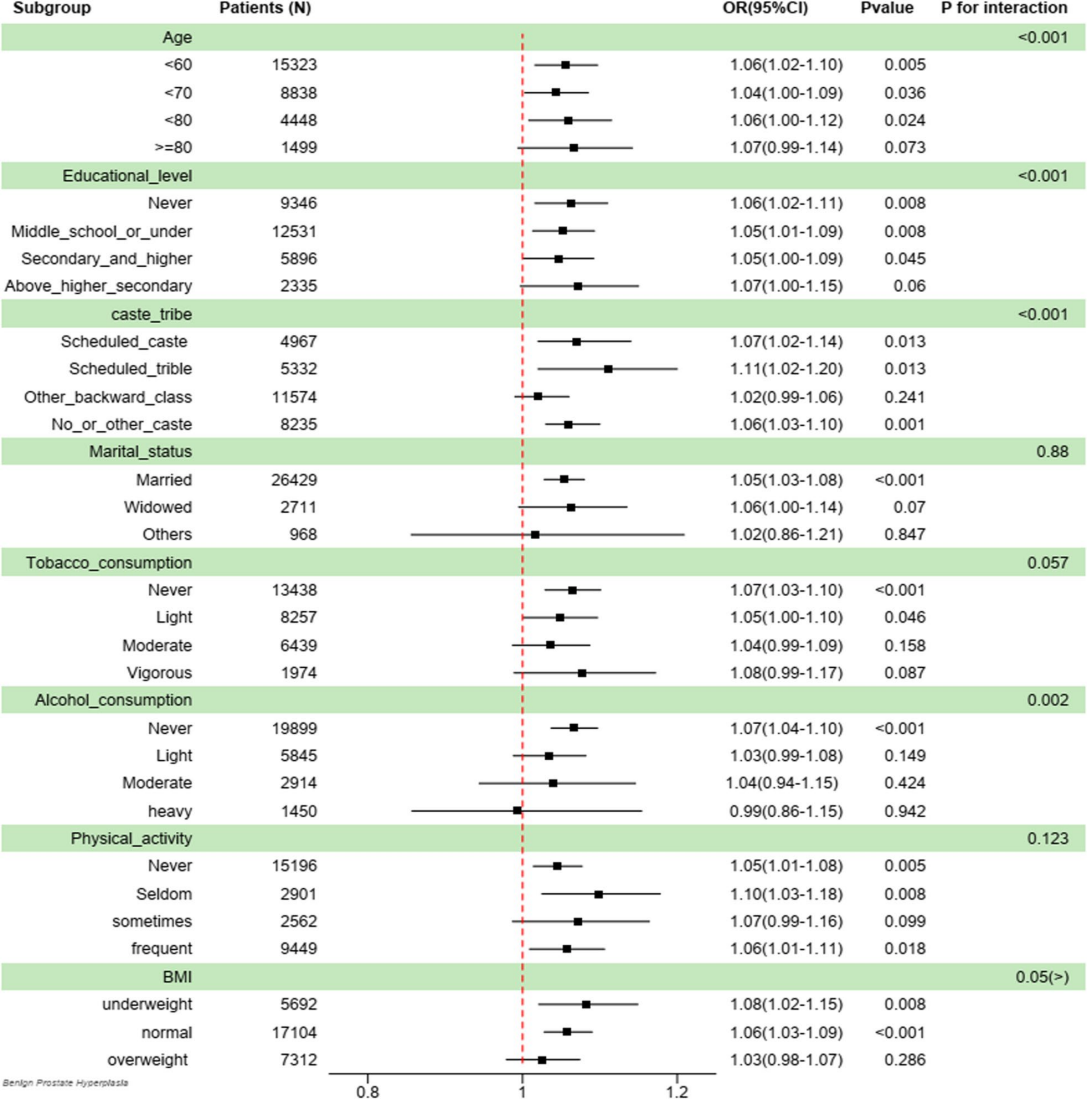
Subgroup analysis between depression and BPH; OR: odds ratio; 95% CI: 95% Confidence interval; BMI: body mass index; underweight: BMI < 18.5 kg/m2; normal: 18.5 kg/m2 ≤ BMI < 25 kg/m2; overweight: BMI ≥ 25 kg/m2

The findings from Fig. 2 indicate a significant correlation between depression score and the likelihood of BPH among the age segment, with the exception of those aged above 80. Furthermore, this correlation remained consistent across various demographic groups, including those with no education, middle school and under, secondary and higher secondary, scheduled caste/tribe and no or other caste. It also held consistent for individuals who were married, never or light tobacco consumption; never alcohol consumption; never, seldom, or frequent physical activity; and normal or underweight BMI groups. The results of the interaction test indicate that there is a significant impact of age, education level, caste/tribe, and alcohol consumption on the link between depression and BPH, which is supported by a statistically significant *p*-value for interaction.

## Discussion

Currently, the worldwide trend toward aging is intensifying, with a growing percentage of the population being elderly [11]. As a large population with a severe aging problem, India is seeing a significant increase in its population, with a notable rise in the proportion of those aged 60 and over projected to occur between 2001 and 2031. This demographic shift is expected to result in an almost twofold increase, reaching around 20 percent by the middle of the century [16]. Common co-occurrence of depression and BPH in the elderly population necessitates additional research to confirm the validity of their association. The LASI, India's first research on aging conducted in 2017–2018 to investigate the physiological, economic, social, and psychological aspects of India's aging process, allowed us to explore the connection between these factors.

Numerous studies have examined the detrimental impact of BPH on quality of life, mainly about general health status and mental health [17, 18]. These studies consistently showed a significant amount of psychiatric morbidity among BPH patients. However, there are few studies on the reverse relationship between depression and BPH, which merits investigation. Several prior studies have established a correlation between depression and BPH symptoms [11, 17, 19], but their findings are difficult to generalize due to many drawbacks. Johnson et al. [19] found that male individuals with depression had American Urological Association Symptom Index scores that were 156% higher than patients without depression. However, they only took into account socioeconomic confounding variables such as education level and income, ignoring other clinical factors that cause LUTS. In addition, due to the statistical design, the cohort size of this study was limited, which might lead to a certain deviation in the conclusion. A prospective cohort investigation conducted by Xiong et al. [11] demonstrated that depressed participants in 2011 displayed higher risks of suffering LUTS/BPH in 2013 than the nondepressed group in a 1351 matched follow-up study. However, they also only adjusted for limited socioeconomic confounding factors such as age and education level, not considering patients’ physical status and history of chronic diseases. Patients with moderate to severe depression (compared to those reporting little depression) had a greater risk of experiencing lower urinary tract symptoms, according to a short-term prospective research done by Chung et al. on 2,890 Chinese males older than 65 years old [17]. However, this study only included elderly men aged ≥ 65 years, and the relationship between depression and prostatic hyperplasia in middle-aged men has not been explored.

Using LASI data, we analyzed the association between depression and BPH in middle- and elderly-aged adults. This research is the broadest population-based investigation on the link between depression and BPH in older individuals in low- and middle-income countries (LAMICs) in recent years. Furthermore, we took into account age, caste, residence, marital status, level of education, tobacco consumption, alcohol consumption, physical activity, body mass index, and a number of other chronic diseases as confounders in our analysis of the association between depression and BPH. Middle-aged and older people with more serious depressive symptoms were more likely to have BPH. After controlling for these possible confounding variables, there was still an association between depression and BPH, which may be connected to age and other diseases.

Although this study confirmed a link between depression and BPH, the mechanism behind this relationship is still unclear. This association may be a result of hormone metabolism and systemic inflammation. According to Gold's research [9], patients with depression have a dysregulated CRH/HPA axis, and hypersecretion of CRH can cause sustained hypercortisolism and excessive activation of the sympathetic nervous system, both of which exacerbate lower urinary tract symptoms due to BPH by affecting urethral smooth muscle via sympathetic innervation [20]. Systemic inflammation is another crucial potential mechanism. Previous research has demonstrated that major depressive disorder patients are in a proinflammatory state, with elevated levels of innate immune plasma mediators such as IL-6, TNF-a, and C-reactive protein [8, 9, 21]. These substances of general inflammation can end up with the proliferation of epithelial and stromal prostatic cells, followed by BPH [22]. Consequently, systemic inflammation, which mediates the association between depression and BPH, might contribute to an increase in BPH prevalence rate.

 Although this research was conducted on a nationwide investigation, it did have a few limitations. Firstly, the majority of BPH diagnoses were based on self-report instead off more objective examinations such as prostatic ultrasonography, which might impact the diagnosis. As this was a cross-sectional study, it is plausible that the causal association between BPH and depression was not precisely determined; however, forthcoming prospective and intervention research could give a more comprehensive explanation. Lastly, certain covariables may be susceptible to recollection bias.

## Conclusions

Our research found that BPH was significantly linked to the presence of depressive symptoms among middle-aged and elderly Indian men. Given the tremendous pace of population aging, it is essential that male individuals who are experiencing symptoms of depression prioritize their prostate health now in order to ensure their life quality. Furthermore, our research also demonstrated that middle-aged and older men with depressive symptoms had an increased likelihood of developing BPH. In addition, further prospective studies are necessary to elucidate this correlation and explore potential mechanisms.

### Supplementary Information

**Additional file 1.**

## Data Availability

Publicly available datasets were analyzed in this study, which can be found at: https://www.iipsindia.ac.in/content/data-request. And the sorted data was provided as Table S1: Supplementary data.
